# A new risk-assessment tool for venous thromboembolism in advanced lung cancer: a prospective, observational study

**DOI:** 10.1186/s13045-022-01259-7

**Published:** 2022-04-04

**Authors:** Yukari Tsubata, Takamasa Hotta, Kosuke Hamai, Naoki Furuya, Toshihide Yokoyama, Ryota Saito, Atsushi Nakamura, Takeshi Masuda, Megumi Hamaguchi, Shoichi Kuyama, Ryoichi Honda, Tadashi Senoo, Masamoto Nakanishi, Masahiro Yamasaki, Nobuhisa Ishikawa, Kazunori Fujitaka, Tetsuya Kubota, Kunihiko Kobayashi, Takeshi Isobe

**Affiliations:** 1 Department of Internal Medicine, Division of Medical Oncology and Respiratory Medicine, Shimane University Faculty of Medicine, 89-1 Enya-cho, Izumo, Shimane 693-8501 Japan; 2 Department of Respiratory Medicine, Hiroshima Prefectural Hospital, 1-5-54 Ujina-kanda, Minami-ku, Hiroshima, 734-8530 Japan; 3 Division of Respiratory Medicine, Department of Internal Medicine, St. Marianna University School of Medicine, 2-16-1 Sugao, Miyamae-ku, Kawasaki, Kanagawa 216-8511 Japan; 4 Department of Respiratory Medicine, Kurashiki Central Hospital, 1-1-1, Miwa, Kurashiki, Okayama 710-8602 Japan; 5 Department of Respiratory Medicine, Tohoku University, 1-1 Seiryo-machi, Aoba-ku, Sendai, Miyagi 980-8574 Japan; 6 Department of Pulmonary Medicine, Sendai Kousei Hospital, 4-15 Hirose-machi, Aoba-ku, Sendai, Miyagi 980-0873 Japan; 7 Department of Respiratory Medicine, Hiroshima University Hospital, 1-2-3 Kasumi, Minami-ku, Hirosima, 734-8553 Japan; 8 Department of Respiratory Medicine, Iwakuni Clinical Center, 1-1-1 Atago-machi, Iwakuni, Yamaguchi 740-8510 Japan; 9 Department of Respiratory Medicine, Asahi General Hospital, I-1326 Asahi, Chiba, 289-2511 Japan; 10 Department of Respiratory Medicine, National Hospital Organization, Kure Medical Center, 3-1 Aoyamacho, Kure, Hiroshima 737-0023 Japan; 11 Department of Medical Oncology, Yamaguchi-Ube Medical Center, 685 Higashikiwa, Ube, Yamaguchi 755-0241 Japan; 12 Department of Respiratory Disease, Hiroshima Red Cross Hospital and Atomic-Bomb Survivors Hospital, 1-9-6, Senda-machi, Naka-ku, Hiroshima, 730-8619 Japan; 13 Department of Respiratory Medicine and Allergology, Kochi University Hospital, 185-1 Kohasu, Oko-cho, Nankoku, Kochi 783-8505 Japan; 14 Department of Pulmonary Medicine, Saitama Medical University International Medical Center, 1397-1 Yamane, Hidaka, Saitama 350-1298 Japan

**Keywords:** Deep vein thrombosis, Pulmonary thromboembolism, Lung cancer, Risk-assessment model, Prothrombin fragment 1 + 2

## Abstract

Management of cancer-associated venous thromboembolism (VTE) is essential in treatment selection and cancer prognosis. However, to date, there is no method to assess the risk of VTE specifically associated with advanced lung cancer. Our aim was to create a new risk assessment scoring system that can predict the concomitant or incidence of VTE in advanced lung cancer. We used the dataset of 1008 patients with lung cancer in the Rising-VTE/NEJ037 study, of which 100 (9.9%) developed VTE. The items extracted in the multivariate analysis included female sex, adenocarcinoma, performance status, N factor, lymphocyte count, platelet count, prothrombin fragment 1 + 2, and diastolic blood pressure. This model had a maximum score of 8 points, with ≥ 5 points indicating a high risk of VTE. This simple risk-assessment model for VTE complications with advanced lung cancer could help identify cases that required monitoring for VTE.

**Supplementary Information:**

The online version contains supplementary material available at 10.1186/s13045-022-01259-7.

**To the editor**

Venous thromboembolism (VTE) is a common medical complication of cancer treatment, and the risk of developing VTE is particularly high in lung cancer patients [1]. Numerous risk score tools to evaluate cancer-associated VTE have been proposed [2, 3]. The Khorana score [2] is the most widely used risk assessment tool for patients scheduled to receive chemotherapy. A meta-analysis reported that the performance of the Khorana score for lung cancer differed from that for other types of cancer and that it was not useful in predicting VTE in lung cancer [4]. As the efficacy of advanced and personalized lung cancer treatments can be maximized by optimally managing complications, such as VTE, there is an urgent need to establish a VTE risk assessment scoring system for lung cancer patients scheduled to receive chemotherapy.

The Rising-VTE/NEJ037 Study, a physician-led, multicenter, prospective, observational study, attempted to identify the incidence of VTE and its risk factors while treating lung cancers for which radical treatments were unsuitable (manuscript in preparation). To our knowledge, the Rising-VTE/NEJ037 Study is the largest prospective study involving intensive screening programs for VTE at the time of cancer diagnosis, along with a further follow-up to assess the incidence of VTE. As many cases of VTE co-developing with lung cancer are asymptomatic, an appropriate risk-assessment scoring system is essential to identify the types of patients who should undergo aggressive screening and monitoring. Here, we describe a newly created risk-assessment scoring system that can predict the co-development or incidence of VTE in advanced lung cancers using the Rising-VTE/NEJ037 Study dataset.

The Rising-VTE/NEJ037 Study included 1008 patients comprising the whole analysis set diagnosed with lung cancer unsuitable for radical resection or radiation between June 2016 and August 2018 across 35 institutions in Japan. The parameters used for risk assessment included age, sex, body mass index, histological classification of the cancer, TNM factors, performance status scores, past medical history, comorbidities, complete blood cell count, coagulation markers (D-dimer, prothrombin fragment 1 + 2 [PT F1 + 2]), liver function markers, kidney function markers, electrolyte levels, C-reactive protein levels, brain natriuretic peptide levels, oxygen saturation, blood pressure, epidermal growth factor receptor gene mutation status, and anaplastic lymphoma kinase fusion gene. We performed a multivariate analysis by logistic regression analysis using a stepwise method to extract the relevant risk factors for VTE. Candidate factors were extracted, and a tenfold cross-validation was used to create a risk-assessment scoring system that ensured internal validity. Receiver operating characteristic (ROC) analysis was performed to estimate the respective cut-off values for each item in the scoring process. The eight risk factors identified by multivariate analysis were evaluated in the ROC analysis, and cut-off values were set (Table 1). The ROC AUC (0.751) indicated a sufficient discriminating ability (Fig. 1).

**Table 1:** New risk scoring system created from the extracted VTE risk factors

Parameter	Coefficient	OR	95% CI	*p*-value	Score point
Overview of the risk score					
Sex, Female (vs. Male)	0.730	2.076	1.257–3.429	0.004	1
Adenocarcinoma (vs. non-small cell lung cancer, others)	0.845	2.327	1.277–4.241	0.006	1
N type, 3 (vs. 0–2)	0.754	2.125	1.299–3.475	0.003	1
Eastern Co-operative Oncology group performance status, 1–3 (vs. 0)	0.754	2.125	1.220–3.704	0.008	1
Lymphocyte percentage < 18%	0.763	2.145	1.293–3.557	0.003	1
Platelet count < 280,000/μL	0.747	2.110	1.238–3.595	0.006	1
Prothrombin fragment 1 + 2 ≥ 325 pmol/L	0.768	2.155	1.313–3.535	0.002	1
Diastolic blood pressure ≥ 70 mmHg	0.658	1.931	1.122–3.325	0.018	1

Points calculated from score	VTE incidence rate
Estimation of the VTE incidence rate using the new risk-assessment scoring system	
0	0.003
1	0.007
2	0.015
3	0.031
4	0.064
5	0.127
6	0.237
7	0.398
8	0.584

Cut-off values for lymphocyte percentage, platelet count, prothrombin fragment 1 + 2 level, and diastolic blood pressure were estimated by ROC analysis

As VTE was observed in 100 of the 1008 cases (incidence rate: 0.099) in the Rising-VTE/NEJ037 Study, scores ≥ 5 were classified into the high-risk group

VTE, venous thromboembolism; CI, confidence interval; OR, odds ratio

**Fig. 1:**
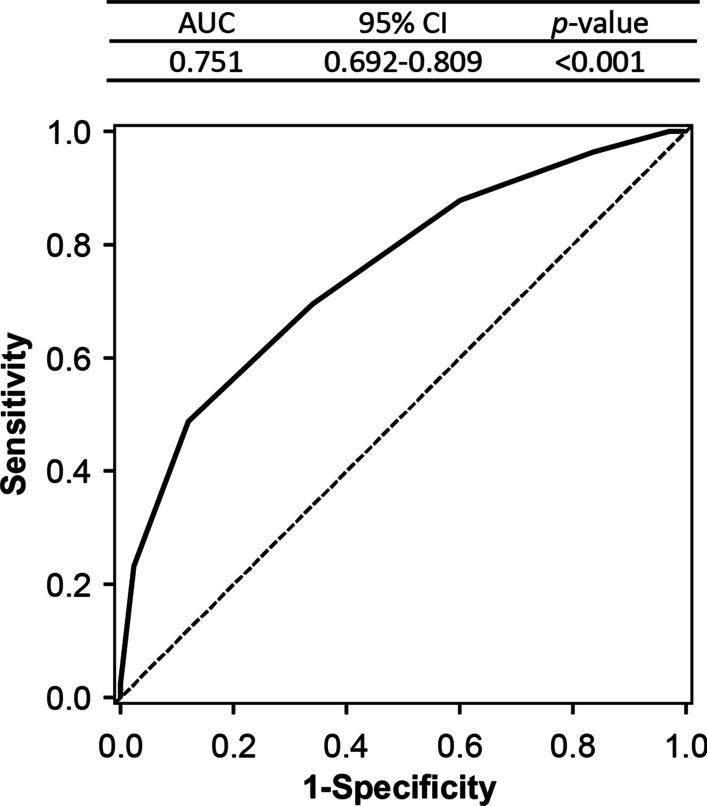
Evaluation of the discriminating ability of the new VTE risk scoring system (receiver operating characteristic curve)

To our knowledge, this is the first study to show that low PLT counts and elevated DBP are risk factors for VTE. Additionally, we revealed that an elevated D-dimer level is not a risk factor and that PT F1 + 2 is a more suitable serum marker involved in coagulation for risk identification. PT F1 + 2 has been reported to be particularly useful as a predictor of cancer-associated thrombosis when used in combination with D-dimer [5]; its usefulness should be verified in future studies.

As cancer-related VTEs are often asymptomatic, risk scores that help actively screen patients at high risk of developing VTE are clinically important. Furthermore, identifying patient populations at a high risk of developing VTE using a thoroughly tested risk-assessment scoring system can balance the complications from adverse events, such as bleeding, with the benefits of prophylactic treatments administered for VTE in patients scheduled to receive chemotherapy. Therefore, our proposed predictive scoring system for the risk of VTE onset in advanced lung cancers may have great value in clinical settings.

This study had some limitations. Whether our proposed risk scoring system would be useful in non-Japanese patients should be examined. In addition, although it underwent internal validation, external validation by other studies is required. We expect that with an increase in the number of cancer patients achieving long-term survival, there will be a greater focus on the diagnosis and treatment of VTE co-developing with cancer in the future.

## Supplementary Information

**Additional file 1**. Supplemental methods

**Additional file 2**. Patient characteristics at the time of lung cancer diagnosis

**Additional file 3**. Univariate analysis of VTE risk

**Additional file 4**. Proposed new risk score

## Data Availability

The datasets used and/or analyzed during the current study are available from the corresponding author on reasonable request.

## Abbreviations

AUC: Area under the curve
CRP: C-reactive protein
CT: Computed tomography
DBP: Diastolic blood pressure
DOAC: Direct oral anticoagulant
DVT: Deep-vein thrombosis
EDO: Edoxaban
NCCN: National Comprehensive Cancer Network
PLT: Platelet
PT F1 + 2: Prothrombin fragment 1 + 2
ROC: Receiver operating characteristic
VTE: Venous thromboembolism
