# Supplementary material for: A new risk-assessment tool for venous thromboembolism in advanced lung cancer: a prospective, observational study
Source: J Hematol Oncol. 2022 Apr 4;15:40. doi: 10.1186/s13045-022-01259-7 (PMC8981807; doi:10.1186/s13045-022-01259-7)
Supplement: Supplementary file 1 — Additional file 1. Supplemental methods [file 13045_2022_1259_MOESM1_ESM.docx]

**Additional file 1**

**Methods**

*Patients*

Case enrollment for the Rising-VTE/NEJ037 Study was conducted between June 2016 and August 2018, and patients were followed up for two years until August 2020. The main eligibility criteria were as follows: diagnosis of small cell lung cancer or non-small cell lung cancer based on cytological or histological examinations; an Eastern Co-operative Oncology Group performance status (PS) of 0–3; age ≥20 years at the time of providing consent; small cell lung cancer for which radical surgery, radiotherapy, and chemotherapy were not possible (regardless of whether the disease was categorized as a limited disease or an extensive disease); non-small cell lung cancer for which radical surgery, radiotherapy, and chemotherapy were impossible (regardless of disease stage); postoperative recurrence or disease recurrence after radical radiotherapy; patient conditions were not indicated for aggressive treatments, such as chemotherapy; and an expected survival period of >6 months after consent.

Since the Rising-VTE/NEJ037 Study was an observational study, no exclusion criteria were provided for case enrollment. Written informed consent was obtained from all patients.

Patients who met the eligibility criteria for the Rising-VTE/NEJ037 Study underwent evaluations for VTE co-development by contrast-enhanced computed tomography (CT) of the chest to the lower extremities or contrast-enhanced CT of the chest to the pelvis, along with lower-extremity venous ultrasound. Furthermore, they were classified into either the observation group without VTE co-development or the cancer-associated VTE group. VTE diagnoses were confirmed through a central review by two radiologists.

Patients with deep vein thrombosis (DVT) with proximal DVT (popliteal vein, femoral vein, and iliac vein thrombosis) identified by contrast-enhanced CT or lower-extremity venous ultrasound were diagnosed with DVT requiring treatment and assigned to the edoxaban (EDO) group; those with isolated distal DVT (thrombosis found only in the soleus, sural, posterior tibial, or anterior tibial vein) who were asymptomatic were retested two weeks later using the same testing modality. If they showed enlargement or progression of the proximal DVT, they were diagnosed with DVT requiring treatment and assigned to the EDO group. Lastly, patients who showed thrombotic embolism of the area, lobe artery, or main pulmonary artery on contrast-enhanced CT were also assigned to the EDO group.

*Risk assessment for VTE*

To create the risk-assessment score system, we analyzed the complete background information and clinical data, including results of hematological examinations and imaging data that could confirm the presence of VTE, of patients. The parameters used for risk assessment included age, sex, BMI, histological classification of the cancer, TNM factors, PS scores, past medical history (stroke, myocardial infarction, and other malignant tumors), comorbidities (chronic obstructive pulmonary disease, rheumatoid arthritis, diabetes, hypertension, dyslipidemia, and other malignant tumors), complete blood cell count, coagulation markers (D-dimer, prothrombin fragment 1+2 [PT F1+2]), liver function markers, kidney function markers, electrolyte levels, C-reactive protein (CRP) levels, brain natriuretic peptide (BNP) levels, oxygen saturation (SpO_2_), blood pressure, epidermal growth factor receptor (EGFR) gene mutation status, and anaplastic lymphoma kinase (ALK) fusion gene. All clinical information was extracted from the data available at the time of the lung cancer diagnosis.

*Statistical analysis*

The target sample size of the Rising-VTE/NEJ037 Study aimed to exceed that of the large-scale cohorts reported thus far as the VTE complication rate in Japanese patients with lung cancer was unknown at the time of study planning. Since the prospective cohort trial at that time was on the scale of hundreds of cases, the target sample size of this trial was set to 1,000 cases.

After performing a univariate analysis of each factor, we performed a multivariate analysis by logistic regression analysis using a stepwise method to extract the relevant risk factors for VTE. Candidate factors were extracted, and a 10-fold cross-validation was used to create a risk-assessment score that ensured internal validity. The ROC analysis was performed to estimate the respective cut-off values for each item in the scoring process. All statistical analyses were conducted using SPSS Statistics (version 24.0; IBM Japan, Ltd., Tokyo, Japan).

*Ethics*

This study was conducted in accordance with the principles of the Declaration of Helsinki and the Good Clinical Practice Guidelines. The study protocol was approved by the Shimane University Institutional Review Board (No. 3024) based on the Clinical Trials Act enacted in Japan in 2017 and published in the Japan Registry of Clinical Trials (jRCTs061180025). Written informed consent was obtained from all patients.
