# Supplementary material for: A new risk-assessment tool for venous thromboembolism in advanced lung cancer: a prospective, observational study
Source: J Hematol Oncol. 2022 Apr 4;15:40. doi: 10.1186/s13045-022-01259-7 (PMC8981807; doi:10.1186/s13045-022-01259-7)
Supplement: Supplementary file 2 — Additional file 2. Patient characteristics at the time of lung cancer diagnosis [file 13045_2022_1259_MOESM2_ESM.docx]

**Additional file 2**. Patient characteristics at the time of lung cancer diagnosis.

| **Characteristics** | | **Total**  **(N=1,008)** | **With VTE**  **(n=62)** | **Without VTE**  **(n=946)** | ***p*-value** |
| --- | --- | --- | --- | --- | --- |
| Age (years) | Median | 70 | 70 | 71 | 0.841 |
|  | Range | 30–94 | 41–81 | 30–94 |  |
| Sex, n (%) | Male | 714 (70.8) | 33 (53.2) | 681 (72.0) | 0.005 |
|  | Female | 294 (29.2) | 29 (46.8) | 265 (28.0) |  |
| ECOG PS, n (%) | 0 | 403 (40.0) | 15 (24.2) | 388 (41.0) | 0.001 |
|  | 1 | 490 (40.6) | 35 (56.5) | 455 (48.1) |  |
|  | 2 | 74 (7.3) | 4 (6.5) | 70 (7.4) |  |
|  | 3 | 41 (4.1) | 8 (12.9) | 33 (3.5) |  |
| Histological type, n (%) | Adenocarcinoma | 641 (63.6) | 55 (88.7) | 586 (61.9) | 0.017 |
|  | Squamous | 187 (18.6) | 4 (6.5) | 183 (19.3) |  |
|  | Small cell | 137 (13.6) | 1 (1.6) | 136 (14.4) |  |
|  | Other | 43 (4.3) | 2 (3.2) | 41 (4.3) |  |
| Clinical stage, n (%) | |  |  |  |  |
| T factor | T1 | 160 (16.8) | 9 (16.1) | 151 (16.9) | 0.431 |
|  | T2 | 255 (26.8) | 22 (39.3) | 233 (26.0) |  |
|  | T3 | 213 (22.4) | 8 (14.3) | 205 (22.9) |  |
|  | T4 | 287 (30.1) | 15 (28.8) | 272 (30.4) |  |
|  | Tx | 37 (3.9) | 2(3.6) | 35 (3.9) |  |
|  | Missing | 56 | 6 | 50 |  |
| N factor | N0 | 195 (20.2) | 8 (13.8) | 187 (20.7) | 0.196 |
|  | N1 | 98 (10.2) | 6 (10.3) | 92 (10.2) |  |
|  | N2 | 268 (27.8) | 10 (17.2) | 258 (28.5) |  |
|  | N3 | 402 (41.7) | 34 (58.6) | 368 (40.7) |  |
|  | Missing | 45 | 4 | 41 |  |
| M factor | M0 | 192 (20.0) | 5 (8.6) | 187 (20.7) | 0.024 |
|  | M1a | 228 (23.8) | 9 (15.5) | 219 (24.3) |  |
|  | M1b | 540 (56.3) | 44 (75.9) | 496 (55.0) |  |
|  | Missing | 48 | 4 | 44 |  |

The P-value was calculated using the Kruskal–Wallis or Chi-square method. The P-value of < 0.05 was considered statistically significant.

Clinical stages were assigned according to UICC7 (7th edition TNM staging system for lung cancer).^12^

ECOG PS: Eastern Cooperative Oncology Group performance status.
