# Supplementary material for: A new risk-assessment tool for venous thromboembolism in advanced lung cancer: a prospective, observational study
Source: J Hematol Oncol. 2022 Apr 4;15:40. doi: 10.1186/s13045-022-01259-7 (PMC8981807; doi:10.1186/s13045-022-01259-7)
Supplement: Supplementary file 3 — Additional file 3. Univariate analysis of VTE risk [file 13045_2022_1259_MOESM3_ESM.docx]

**Additional file 3**: Univariate analysis of VTE risk

| **Parameter** | **OR** | **95% CI** | ***p*-value** |
| --- | --- | --- | --- |
| Sex, F (vs. M) | 1.975 | 1.295–3.011 | 0.002 |
| Histological type, small (vs. non-small) | 0.379 | 0.162–0.882 | 0.024 |
| Adenocarcinoma (vs. NSCLC others) | 2.173 | 1.329–3.553 | 0.002 |
| NSCLC_2_Squamous cell carcinoma, 1 (vs. 0) | 0.630 | 0.344–1.155 | 0.135 |
| NSCLC_3_Large cell carcinoma, 1 (vs. 0) | n.c. |  |  |
| NSCLC_99_Other, 1 (vs. 0) | 0.689 | 0.209–2.272 | 0.541 |
| NSCLC_STAGE |  |  |  |
| Ib | n.c. |  |  |
| IIa | n.c. |  |  |
| IIb | n.c. |  |  |
| IIIa | 0.386 | 0.048–3.094 | 0.370 |
| IIIb | 0.446 | 0.122–1.636 | 0.224 |
| IV | 1.491 | 0.788–2.822 | 0.220 |
| Postoperative recurrence | 1.000 | ref |  |
| T factor |  |  |  |
| T1a | 1.000 | ref |  |
| T1b | 0.418 | 0.133–1.317 | 0.136 |
| T2a | 0.497 | 0.169–1.458 | 0.203 |
| T2b | 0.920 | 0.282–2.999 | 0.890 |
| T3 | 0.451 | 0.154–1.321 | 0.146 |
| T4 | 0.439 | 0.154–1.255 | 0.124 |
| TX | 0.558 | 0.135–2.303 | 0.420 |
| N factor |  |  |  |
| 0 | 1.000 | ref |  |
| 1 | 1.542 | 0.627–3.795 | 0.346 |
| 2 | 1.297 | 0.622–2.703 | 0.488 |
| 3 | 2.316 | 1.207–4.443 | 0.012 |
| ECOG PS |  |  |  |
| 0 | 1.000 | ref |  |
| 1 | 2.070 | 1.271–3.371 | 0.003 |
| 2 | 1.833 | 0.793–4.237 | 0.157 |
| 3 | 3.665 | 1.533–8.767 | 0.004 |
| Comorbidity |  |  |  |
| COPD | 0.769 | 0.446–1.327 | 0.345 |
| RA_Rheumatoid arthritis | 1.524 | 0.336–6.908 | 0.585 |
| DM | 0.500 | 0.262–0.955 | 0.036 |
| Malignant tumor | 0.391 | 0.094–1.638 | 0.199 |
| Hypertension | 0.861 | 0.564–1.314 | 0.487 |
| Dyslipidemia | 0.879 | 0.526–1.471 | 0.624 |
| Other | 0.764 | 0.492–1.188 | 0.232 |
| Medical history |  |  |  |
| Stroke | 0.918 | 0.386–2.185 | 0.848 |
| Myocardial infarction | 2.120 | 0.785–5.726 | 0.138 |
| Malignant tumor | 0.620 | 0.315–1.221 | 0.167 |
| Other, 1 (vs. 0) | 0.807 | 0.515–1.264 | 0.349 |
| AGE (per 1) | 0.992 | 0.972–1.013 | 0.468 |
| WBC (per 1/μL) | 1.000 | 1.000–1.000 | 0.976 |
| NEUT (per 1/µL) | 1.000 | 1.000–1.000 | 0.554 |
| EOS (per 1%) | 0.932 | 0.849–1.024 | 0.142 |
| BASO (per 1%) | 0.561 | 0.304–1.035 | 0.064 |
| MONO (per 1%) | 0.954 | 0.867–1.050 | 0.339 |
| LYMPH (per 1%) | 0.969 | 0.946–0.993 | 0.011 |
| Hemoglobin (per 1 g/dL) | 0.972 | 0.861–1.097 | 0.643 |
| PLT (per 10000/μL) | 0.975 | 0.953–0.997 | 0.027 |
| PT-INR (per 1sec) | 0.890 | 0.260–3.046 | 0.853 |
| APTT (per 1%) | 0.952 | 0.906–1.000 | 0.050 |
| D-dimer (per 5 μg/mL) | 1.232 | 1.111–1.365 | 0.000 |
| PT F1+2 (per 50 pmol/L) | 1.120 | 1.073–1.169 | 0.000 |
| Total protein (per 1g/dL) | 0.697 | 0.495–0.982 | 0.039 |
| ALB (per 1 g/dL) | 0.859 | 0.603–1.224 | 0.400 |
| LDH (per 1U/L) | 1.000 | 1.000–1.001 | 0.273 |
| AST (per 1U/L) | 1.001 | 0.992–1.010 | 0.858 |
| ALT (per 1U/L) | 1.001 | 0.991–1.011 | 0.855 |
| BUN (per 1 mg/dL) | 0.979 | 0.937–1.023 | 0.343 |
| Crea (per 1 mg/dL) | 0.352 | 0.112–1.110 | 0.075 |
| T-Bil (per 1 mg/dL) | 0.884 | 0.521–1.500 | 0.648 |
| NA (per 1 mEq/L) | 0.998 | 0.940–1.059 | 0.950 |
| K (per 1 mEq/L) | 1.099 | 0.668–1.810 | 0.710 |
| CL (per 1 mEq/L) | 1.010 | 0.956–1.066 | 0.729 |
| CRP (per 1 mg/dL) | 1.001 | 0.954–1.049 | 0.978 |
| BNP (per 1 pg/mL) | 1.001 | 0.999–1.003 | 0.187 |
| CCR (per 1 mL/min) | 1.003 | 0.995–1.011 | 0.481 |
| SPO_2_ (per 1%) | 0.892 | 0.804–0.988 | 0.029 |
| sBP (per 1 mmHg) | 1.008 | 0.995–1.020 | 0.235 |
| dBP (per 1mmHg) | 1.019 | 1.000–1.038 | 0.049 |

OR: odds ratio; 95% CI: 95% confidence interval; ref: reference; n.c.: cannot be calculated.
