# Supplementary material for: A new risk-assessment tool for venous thromboembolism in advanced lung cancer: a prospective, observational study
Source: J Hematol Oncol. 2022 Apr 4;15:40. doi: 10.1186/s13045-022-01259-7 (PMC8981807; doi:10.1186/s13045-022-01259-7)
Supplement: Supplementary file 4 — Additional file 4. Proposed new risk score [file 13045_2022_1259_MOESM4_ESM.docx]

**Additional File 4.** Proposed new risk score

| Parameter | Criterion | Score point |
| --- | --- | --- |
| Sex | Female | 1 |
| Histology | Adenocarcinoma | 1 |
| N type | 3 | 1 |
| ECOG PS | 1–3 | 1 |
| Lymphocyte percentage | <18% | 1 |
| Platelet count | <280,000/μL | 1 |
| Prothrombin fragment 1 + 2 | ≥325 pmol/L | 1 |
| Diastolic blood pressure | ≥70 mmHg | 1 |

ECOG PS, Eastern Cooperative Oncology Group performance status
